# Preschool attention and sleep support (PASS): protocol for a pilot feasibility randomized clinical trial

**DOI:** 10.3389/frsle.2025.1662221

**Published:** 2026-02-06

**Authors:** Naomi O. Davis, Brian Eichner, Matthew J. Gibson, Jessica R. Lunsford-Avery

**Affiliations:** 1 Department of Psychiatry and Behavioral Sciences, Duke University School of Medicine, Durham, NC, United States; 2 Department of Pediatrics, Duke University School of Medicine, Durham, NC, United States

**Keywords:** sleep, ADHD, preschool, behavioral therapy, behavioral sleep medicine, parent management training

## Abstract

**Introduction:**

Attention-Deficit/Hyperactivity Disorder (ADHD) symptoms often emerge during preschool, highlighting a critical period for prevention. Preventative ADHD interventions may be most effective if they target biological mechanisms linked to core ADHD pathophysiology. Sleep dysregulation represents a potential target, yet the gold-standard behavioral intervention (behavioral parent training, BPT) focuses primarily on ameliorating daytime impairment. There is a critical need to adapt BPT to target behaviors across the 24-h period through integration with behavioral sleep medicine (BSM).

**Methods:**

This trial will randomize children ages 3–5 years who are identified as at-risk for ADHD (i.e., with elevated ADHD symptoms) and their caregivers to receive either BPT (*n* = 22) or a combined intervention that includes BPT and BSM (Preschool Attention and Sleep Support, PASS; *n* = 22). Blinded assessments will be conducted at baseline, immediately post-treatment, and 3 months post treatment. Feasibility and acceptability will be assessed.

**Results:**

Key outcomes will include changes in ADHD symptoms (measured by clinical and caregiver rating) and sleep (measured by both actigraphy and caregiver report). Changes in additional functional outcomes (e.g., comorbid symptoms, parenting stress) will be explored.

**Discussion:**

Findings from this study will provide essential data to inform a large-scale clinical trial of PASS, with the ultimate goal of improving functional outcomes among preschoolers at risk for ADHD and modifying the trajectory of this chronic condition through early preventative intervention focused on improving biological processes linked to ADHD.

**Trial Registration:**

NCT05862727.

## 1 Introduction

ADHD is neurodevelopmental disorder that emerges in childhood, with current prevalence estimated at about 10% of youth (Xu et al., 2018). Onset of symptoms during the preschool years may reflect particular risk for adverse outcomes (Childress and Stark, 2018), including persistent challenges with core ADHD symptoms (McGee et al., 1991), social and academic impairment (Lahey et al., 2016; Sjowall et al., 2017), cognitive deficits (McGee et al., 1991), and general family distress and economic burden (Doshi et al., 2012; Zhao et al., 2019; Libutzki et al., 2019; American Psychiatric Association, 2013). Most children are not identified with ADHD nor receive treatment for ADHD until middle or late childhood, which delays onset of intervention and misses a critical period during which treatment may have a particularly strong impact on improving ADHD trajectories (Sonuga-Barke and Halperin, 2010). As such, a prevention-based approach for young children at-risk for ADHD (i.e., those displaying elevated ADHD symptoms) is indicated (Sonuga-Barke and Halperin, 2010; Sonuga-Barke et al., 2011). The recommended first-line treatment for preschoolers at-risk for or diagnosed with ADHD is behavioral parent training (BPT) (Wolraich et al., 2019). BPT has a robust empirical basis for ADHD-related concerns (i.e., disruptive behaviors, parent stress and competence), but treatment effects are inconsistently found for core ADHD symptoms (i.e., inattention, hyperactivity/impulsivity) (Antshel and Barkley, 2008) and rarely generalize to other settings (Sonuga-Barke et al., 2013; Abikoff et al., 2015).

Preventive interventions for ADHD may be most effective if they target biological mechanisms linked to core ADHD pathophysiology (Sonuga-Barke and Halperin, 2010). Sleep dysregulation is a candidate biological process that demonstrates a strong association with ADHD symptoms. Specifically, compared to typically developing peers, preschoolers with elevated ADHD symptoms and/or an ADHD diagnosis display greater sleep problems, longer sleep latency, shorter sleep duration, more night awakenings, and more daytime sleepiness as assessed via parent-report (Scott et al., 2013; Goodlin-Jones et al., 2009; Kaplan et al., 1987; Cao et al., 2018; Willoughby et al., 2008; DeVincent et al., 2007; Hiscock et al., 2007; Van Dyk et al., 2019), as well as greater nocturnal activity, lower daytime activity, and more irregular sleep patterns as measured via actigraphy (Melegari et al., 2020; Bundgaard et al., 2018). Co-occurring sleep dysregulation is related to increased core ADHD symptom severity, poorer cognition and functioning, and risk for internalizing and externalizing disorders in school-aged children (Dimakos et al., 2021; Bondopadhyay et al., 2002). Researchers have posited ADHD and sleep as “two sides of the same coin,” suggesting that eliminating daytime deficits may depend on addressing sleep regulation (Bijlenga et al., 2019). As such, sleep-focused interventions may positively impact ADHD symptom trajectories as well as related impairments (e.g., functional deficits, comorbidity; Lunsford-Avery et al., 2025). Limited work has been conducted to date with preschool-aged children; however, studies of school-aged youth with ADHD indicate that caregiver-focused behavioral sleep medicine (BSM) interventions can improve sleep, core ADHD symptoms, and general psychosocial functioning (Sciberras et al., 2011, 2020; Hiscock et al., 2015; Corkum et al., 2016; Keshavarzi et al., 2014). BSM and BPT interventions are rooted in analogous behavioral theories which emphasize behavior change through (1) caregiver optimization of antecedents of behavior (e.g., providing effective instructions), and (2) operant conditioning, which theorizes that consequences can be modified (e.g., positive rewarding of desired behaviors and removing attention from undesired behaviors) and, in turn, shape behaviors (Abikoff et al., 2015; Barkley, 1998; Mindell and Owens, 2015; Meltzer, 2010). For BPT, the target mechanism is positive parenting, generally for daytime behaviors, which supports the caregiver-child relationship and increases effectiveness of punishments by situating them in a generally positive environment (Abikoff et al., 2015; Barkley, 1998; Haack et al., 2017, 2016; Forehand et al., 2014; Chronis-Tuscano et al., 2011). In contrast, the target mechanism of BSM is sleep regulation, which focuses on nighttime behaviors including setting a consistent sleep schedule, creating a bedtime routine, and teaching the child to fall asleep independently (Mindell and Owens, 2015; Meltzer, 2010; Mindell et al., 2006, 2015; Honaker and Meltzer, 2014; Morgenthaler et al., 2006).

Recognizing the concurrent and prospective links between sleep and core ADHD symptoms, we developed a 9-week telehealth intervention (Preschool Attention and Sleep Support; PASS) that targets behaviors across the 24-h period for preschoolers with elevated ADHD symptoms and sleep dysregulation. Our goal was to develop a single, efficient intervention that extends beyond positive parenting to target sleep regulation as a key mechanism that may be particularly effective for improving sleep and ADHD trajectories across early development and beyond. PASS conceptualizes ADHD as a 24-h disorder and combines two empirically-supported behavioral interventions to address daytime (via BPT), and nighttime (via BSM) ADHD-related behaviors by modifying the environment; setting clear expectations and consistent routines; and employing parental attention to shape behaviors. Our pilot randomized control trial will compare the PASS intervention condition to a BPT-only condition which employs similar behavioral principles in the same number of sessions, but only addresses daytime behaviors. The study's primary aims are: (1) to examine feasibility and acceptability of PASS; (2) to assess preliminary effectiveness of PASS compared to BPT in improving core ADHD symptoms; and (3) to assess the effects of PASS compared to BPT on sleep regulation, and to examine whether improved sleep regulation is associated with reduced ADHD symptoms. Changes in additional functional outcomes (e.g., comorbid symptoms, parenting stress) will be also explored.

## 2 Methods and analysis

### 2.1 Study design

Children aged 3–5 years who are identified as at-risk for ADHD and their caregiver(s) will be recruited from pediatric primary care clinics within a large university medical system and the surrounding community. Caregivers/guardians who are interested in participating will complete an Institutional Review Board-approved phone screen with study staff. Potentially eligible families will then attend an onsite visit where caregivers/guardians will consent to the study and then participate in a screening visit to confirm eligibility. If eligible and interested in enrolling in the study, caregivers will be invited to continue with the visit and to complete additional baseline study assessments. For the next 7 days, children will wear an actigraph device (24 h/day) and caregivers will complete an electronic daily diary about their child's sleep (Honaker and Meltzer, 2014). Actigraphy will be scheduled during a “typical” week for the child (e.g., no travel vacations). Following the seventh night of actigraphy, families will attend a brief follow-up visit during which they will return the actigraph and will be randomized to participate in either the PASS or BPT intervention conditions.

**Figure 1:**
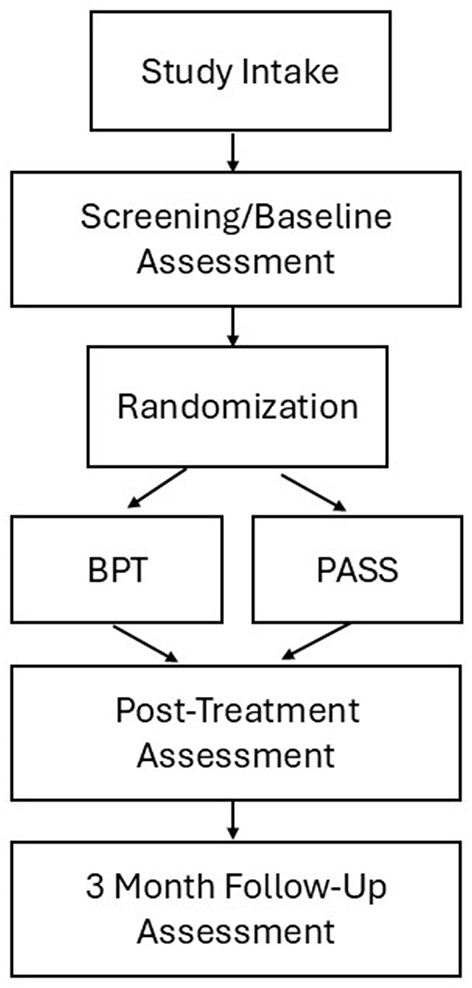
Study design. Note each assessment time point includes assessment of (1) ADHD symptoms and related impairments, and (2) actigraphy and caregiver-reported sleep measures.

Immediately post-intervention, families will return to the clinic for a second assessment visit and another 7 day/night period of actigraphy data collection. Information regarding feasibility and acceptability of the intervention will also be collected. Families will repeat the assessments and actigraphy at 3 months following the intervention. At each follow-up assessment, psychiatric measures will have both clinician-rated (blind to treatment assignment) and caregiver-reported components. See Figure 1 for study design.

### 2.2 Setting, recruitment, and eligibility criteria

We will recruit children who receive primary care services from a group of university health system-affiliated practices in Durham, North Carolina and the surrounding community. Recruitment methods will include using the electronic health record to help identify potential participants who may meet study criteria, primary care provider referral, family self-referral, and sharing study flyers and information with primary care clinics, surrounding community spaces, and electronically (e.g., websites, social media). Inclusion and exclusion criteria are shown in Table 1. Participant recruitment began in February 2024. Enrollment is expected to continue through summer/fall of 2025 and final follow-up for participants is expected to conclude in December 2025. The study is approved by the institution's Internal Review Board (IRB).

**Table 1:** Study inclusion and exclusion criteria.

**Inclusion criteria**	**Exclusion criteria**
(1) Ages 3–5 years at intake	(1) Suspected obstructive sleep apnea or restless legs syndrome (determined via screening questionnaires; Mindell and Owens, 2015)
(2) Score of 30 on the child sleep habits survey-short form (Owens et al., 2000) and a caregiver rating of child's sleep problems as moderate/severe (Sung et al., 2008)	(2) Current use of stimulant medication, other psychotropics, or medications for sleep (prescription or over-the-counter, including melatonin)
(3) ≥80th percentile on the clinician-rated Attention Deficit Hyperactivity Disorder Rating Scale (ADHD-RS) based on age and sex norms, (McGoey et al., 2007; Marin-Mendez et al., 2019) with at least 4 symptoms in the clinically significant range (scores of 2–3) in either the inattention or hyperactive/impulsive domains (Cohen et al., 2018)	(3) Caregiver report of psychiatric disorder other than ADHD requiring treatment (medication and/or therapy), Autism Spectrum Disorder, or intellectual disability
(4) Caregiver ability to speak, read, and write in English	(4) Severe and/or uncontrolled medical condition (e.g., pulmonary and neurological conditions such as cystic fibrosis and seizure disorder) that would interfere with sleep and/or study participation according to the study team
(5) Access to a device with internet and/or smartphone to access telehealth visits	(5) Caregiver is currently participating in another caregiver-focused training intervention or has previously participated in another caregiver training intervention in the past 6 months
(6) Caregiver ability to follow written and verbal instructions	
(7) Ability and willingness to comply with study procedures	

### 2.3 Randomization

Participants will be randomized 1:1 to either the PASS or BPT conditions using the randomization module within REDCap (Harris et al., 2019, 2009). Given the higher prevalence of ADHD in males compared to females in the target age range (2:1) (Willcutt, 2012) and the potential for symptom profiles to differ across sexes (Hartung et al., 2002), randomization will be stratified by participant sex (Kernan et al., 1999). The randomization scheme will be generated using a random number generator in Excel, and the assignments will be implemented within REDCap.

### 2.4 Sample size

Power analyses were performed to estimate detectable effect sizes. The overall correlation among the Level 1 repeated measures was anticipated to be *r* = 0.80 (Sullivan et al., 2021; Badawy and Radovic, 2020). The between-group difference was assessed using a linear trend increasing from no effect at the first time point [i.e., Cohen's *d* (Barnett et al., 2021) = 0.00] to a medium effect size (*d* = 0.50) at the third time point. Results (RMASS; Waltz et al., 1993; Oei and Shuttlewood, 1999; Borkovec and Nau, 1972) revealed the total sample size of 43 subjects (22 in each group) achieved 80% power to detect a medium effect of Cohen's *d* = 0.50 with 80% power at a significance level of *p* = 0.05. As such, and given the exploratory nature of this study, a sample size of 44 participants is anticipated to be sufficient for examining the strength and direction of intervention effects.

### 2.5 Interventions

Both the experimental intervention (PASS) and control intervention (BPT) will be delivered via telehealth. Consistent with prior BPT and BSM protocols for this age group (Barkley, 1998; Mindell and Owens, 2015), each 60-min session will be implemented solely with caregiver(s) without the child present. This modality overcomes many systemic barriers to accessing behavioral health care for families by increasing flexibility for caregivers, reducing time and travel needed to attend sessions, limiting the need for childcare, and facilitating application of skills in the caregiver's “real world” (e.g., home) setting (Sullivan et al., 2021; Badawy and Radovic, 2020; Barnett et al., 2021). See Table 2 for components for the PASS and BPT conditions.

**Table 2:** Intervention components.

**Components**	**BPT and PASS**	**PASS only**
Target mechanism	Positive parenting	Sleep regulation
Treatment goals	Daytime behavior	Bedtime behavior
Psychoeducation	ADHD, contingency management	Sleep
Specific skill building	ABC framework	Consistent sleep schedule
	Setting clear expectations	Bedtime routines
	Applying positive attention	Sleep hygiene
	Salient rewards	Structured bedtime check-ins
	Effective punishment	Independent sleeping
Parent self-monitoring	Between session practices	Sleep behaviors and goals

#### 2.5.1 PASS

PASS is a 9-week intervention that integrates behavioral interventions for ADHD (behavioral parent training [BPT]; Barkley, 1998) and sleep dysregulation (behavioral sleep medicine; Mindell and Owens, 2015), both of which are well-established and efficacious for use with caregivers of preschoolers (Childress and Stark, 2018; Honaker and Meltzer, 2014). The first four sessions are comprised of evidence-based BPT skills training. Skills address ways to improve daytime behaviors through enhancement of positive parenting skills, including modifying antecedents, applying and withdrawing positive attention as a consequence, and shaping behavior using salient rewards (e.g., token economy system) and effective punishments (e.g., time out) (Barkley, 1998). Sessions 5–8 include evidence-based BSM which aligns with the three primary BSM targets for this age group (i.e., consistent sleep schedule with age-appropriate bedtime, consistent bedtime routine, teaching child to sleep independently; Mindell and Owens, 2015; Meltzer, 2010). Each BSM skill is explicitly tied to a previously learned BPT skill, such that caregivers learn to generalize the same skills to target both daytime and nighttime behaviors. For example, the token economy (i.e., reward system) is initially presented and implemented to address daytime behaviors (e.g., completing an age-appropriate chore) and is later re-introduced as a strategy to reinforce a nighttime behavior (e.g., compliance with bedtime routine). A final session (session 9) is offered as a review and “wrap-up” session. No new content is introduced, but instead caregivers have an opportunity to review previously presented content, problem-solve challenges with skill use, and support the family in setting goals for future use of intervention skills. See Table 3 for a description of PASS session topics.

**Table 3:** PASS and BPT session topics.

**Session**	**Topics covered in both interventions**	
1	Introduction: psychoeducation; principles of behavior management; antecedent-behavior-consequence (ABC) framework; goal setting	
2	Strategic use of parental attention: specific praise and social rewards for positive behaviors; active ignoring of undesirable behaviors	
3	Setting up the child for success: setting clear expectations; giving effective instructions; principles of setting up a reward system (i.e., “token economy”): how to implement and guidelines for appropriate rewards	
4	Reducing negative behaviors: creating “no” or “don't” rules that are clear, concise, and warrant immediate punishment; labeling rule violations; defining punishment as a consequence that decreases a behavior; procedures for effective time out	
	Topics covered in BPT	Topics covered in PASS
5	Psychoeducation about skill application across settings • Review of ABC framework and prior skills • Psychoeducation about ADHD symptoms across settings importance of consistency in applying skills • Goal setting around 3 settings (Meal Times, Play Time, Public Places)	Psychoeducation about sleep support • Sleep education and application of ABC framework • Goal setting about sleep behaviors • Setting a consistent, age-appropriate sleep schedule that allows for adequate duration • For late sleep onset, consider bedtime fading
6	Managing behaviors during meal times • Creating standardized expectations for meal times • Effective instructions/clear rules for meal times • Incorporation of adherence to meal time expectations into token economy	Application of clear expectations to bedtime • Creating a standardized bedtime routine • Effective instructions/clear rules for bedtime behaviors • Sleep hygiene and creating a sleep-conducive environment
7	Managing behaviors during play time • Planning ahead to ensure child appropriate play activities • Strategies to reduce child's interruptions of others • Specific praise and social rewards for prosocial play behaviors	Application of parental attention to bedtime • Specific praise and social rewards for bedtime behaviors • Incorporation of adherence to bedtime routine into token economy
8	Managing behaviors in public places Creating rules for behavior in public places; planning ahead for approved child activities; setting up incentives for compliance and punishments for non-compliance	Application of ignoring to independent sleeping Ignoring bedtime complaints/protests; structured check-ins; bedtime pass; If co-sleeping, consider graduated extinction from parent presence at bedtime
9	Wrap up session	Wrap up session

#### 2.5.2 BPT

The first four sessions of BPT are comprised of the same content as the PASS condition, specifically evidence-based BPT skills training to improve daytime behaviors. Sessions 5–8 focus on practice/application of previously learned skills from the initial sessions. Specifically, each session will focus on applying BPT skills to age-expected settings and situations including behavior (1) at meal times, (2) during play time, and (3) in public places. BPT session topics are distinct from the PASS condition (i.e., do not address bedtimes/sleep) and do not include new BPT skills that are not included in the PASS condition. As with the PASS condition, a ninth session is offered as a review and “wrap-up” session with no new content. See Table 3 for a description of BPT session topics.

### 2.6 Interventionist training and fidelity

PASS and BPT intervention sessions will be delivered by study therapists who are behavioral health providers with clinical practices in the university health system. These therapists will have general experience working with families and applying behavioral treatment principles for children, but no prior experience with BSM and will not be members of the research team that developed the intervention. Study therapists will receive intervention-specific education and training in both the PASS and BPT conditions which will include didactic presentation of each element of the intervention, demonstration of techniques, and role-play of common scenarios. After completion of structured training, intervention implementation will be monitored through weekly supervision which will include review of audio recordings of sessions to support implementation fidelity. Feedback on implementation will be provided. To reduce potential contamination across study conditions, therapists will receive explicit training and instruction about the common elements of PASS and BPT vs. the components specific to each intervention. Therapist training will also address the potential for condition contamination; specific methods to avoid contamination (i.e., adherence to the detailed session outline for each session); and specific strategies to employ if caregivers request content specific to the other intervention (e.g., redirection to content appropriate to the randomized condition). These concepts will be reinforced each week during supervision to minimize drift.

Every telehealth intervention session will be recorded to allow for assessment of intervention fidelity. A checklist for each session will be created that lists each session's content and practices. The checklists will evaluate whether each session component was delivered (yes/no). This checklist will also list items that are specific to the other treatment condition, and raters will be asked to evaluate whether each of the components was delivered (yes/no). In this context, a response of “yes” will indicate contamination has occurred. Raters will attain 80% agreement on training transcripts before beginning fidelity ratings. Raters will code 33% of session recordings (randomly selected). At least 75% protocol adherence (Waltz et al., 1993) will be used to establish intervention fidelity.

### 2.7 Outcome measures

Please see Table 4 for an overview of all outcome measures.

**Table 4:** Overview of outcomes and measures at each time point.

**Outcome/measure**	**Screen**	**Pre**	**Treatment**	**Post**	**FU**
Feasibility and acceptability metrics					
Treatment fidelity			S		
Recruitment	S				
Randomization		S			
Treatment differentiation			S		
Integrity of blinding				A, C	
Attendance			T		
Adherence			T		
Retention				S	S
Treatment satisfaction/credibility				C	
ADHD and behavioral outcomes					
ADHD rating scale	A, C			A, C	A, C
Impairment rating scale		A		A	A
Child behavior checklist		C		C	C
Parental stress scale		C		C	C
Parenting sense of competence		C		C	C
Home situations questionnaire		C		C	C
Sleep outcomes					
Child sleep habits questionnaire	C			C	C
Actigraphy		CP		CP	CP

S, study staff; T, therapist; C, caregiver; A, assessor (blinded); CP, child participant; Screen, screening visit; Pre, pre-treatment Assessment; Post, post-treatment assessment; FU, 3 month follow up.

#### 2.7.1 Feasibility

We will examine feasibility of recruitment and randomization, as well as maintaining integrity of the blinded control intervention. Feasibility of recruitment will be determined by ≥50% enrollment of those who attend a screening visit. Feasibility of randomization will be determined by ≥75% randomization of those who meet study eligibility after attending the screening visit. Integrity of the control condition will be measured by: (1) treatment differentiation (i.e., establishing that no core elements of PASS were administered in the BPT group via administration of the PASS fidelity checklist in the control condition) and (2) integrity of the blind by asking all blinded informants (i.e., caregivers, study assessor) to guess whether the child and family received the experimental or control intervention and to state their confidence in this guess on a 1–10 scale.

#### 2.7.2 Acceptability

Participant attendance will serve as an index of treatment engagement, with a benchmark set at an average attendance of ≥75% of sessions for the PASS condition. Adherence to between-session practice will be assessed via a therapist rating immediately after each session assessing the degree to which the caregiver was adherent overall with homework on a scale of 1 (not at all) to 5 (extremely strong). Retention will be measured by the number of families who complete post-treatment and follow-up assessments and will be determined by 90% completion of post-treatment data collection and 80% completion of follow-up assessments.

We will examine PASS acceptability with the Satisfaction with Therapy and Therapist Scale (STTS-R) (Oei and Shuttlewood, 1999). Caregivers will rate 12 items that assess satisfaction with the therapist and the intervention on a 5-point Likert-type scale from 1 (strongly disagree) to 5 (strongly agree). Higher scores indicate greater satisfaction. Treatment credibility will be measured using a 4-item adaptation of the Client Credibility Questionnaire (Borkovec and Nau, 1972; Silverman et al., 1999). Caregivers rate how logical they found treatment and how confident they were in the treatment on a 9-point scale. High scores indicate stronger credibility.

#### 2.7.3 ADHD outcome measures

The primary outcome of ADHD symptoms will be assessed via the Attention-Deficit/Hyperactivity Disorder Rating Scale, Preschool Version (ADHD-RS), which is a commonly used measure of ADHD symptom severity in both observational studies and clinical trials with preschoolers (McGoey et al., 2007). The ADHD-RS can be completed by clinicians via interview and caregivers via self-report. Similar to the original measure for school-aged children (DuPaul, 1998), the preschool version is associated with good to excellent internal consistency, test-retest reliability, and criterion validity (McGoey et al., 2007; Marin-Mendez et al., 2019). The ADHD-RS will be completed by a blinded Ph.D. level clinical psychologist assessor, and these clinician ratings will be the primary outcome for this study. Parents will also independently complete the ADHD-RS and these ratings will be a secondary outcome.

#### 2.7.4 Sleep outcome measures

Caregiver ratings of sleep will be gathered via the 23-item Children's Sleep Habits Questionnaire-Short Form (CSHQ-SF) (Bonuck et al., 2017), which is a modified version of the widely used 33-item CSHQ (Owens et al., 2000). The CSHQ-SF assesses 6 of the original CSHQ domains that are “behaviorally-based:” bedtime resistance, sleep onset delay, sleep duration, sleep anxiety, night wakings and daytime sleepiness, and omits two domains that are “medically-based:” parasomnias and sleep disordered breathing. The CSHQ-SF has been shown to function as well as the full CSHQ in discriminating between preschoolers with and without a parent-reported behavioral sleep problem, and with and without prolonged sleep latency per actigraphy (Bonuck et al., 2017). The CSHQ has been frequently used in BSM clinical trials with preschoolers (Mindell et al., 2006; Honaker and Meltzer, 2014) and the short form was selected as it specifically queries domains that can be targeted via brief behavioral intervention (Bonuck et al., 2017).

Objective sleep ratings will be obtained via actigraphy which will be collected using an ActiGraph wGT3X-BT monitor worn on the child's non-dominant wrist. Children will wear the device 24 h/day for each 7-day assessment. The devices are easy to use, acceptable to youth and families, reliable and valid to estimate sleep/wake indices, and commonly used with preschoolers in clinical research settings (Lunsford-Avery et al., 2020; Meltzer et al., 2012; Belanger et al., 2013; Schoch et al., 2021). They are equipped with off-wrist detection, provide long-term recording, enable rapid charging and data retrieval via USB connectivity, and are water-resistant. Data will be collected in 60-s epochs which will be designated sleep/wake using the validated Sadeh algorithm (Sadeh et al., 1994). Actigraphy will be accompanied by a daily, electronic sleep diary in which caregivers will report on their child's previous night bedtime, sleep-onset latency, wake time during the night, time of final awakening, final rising time, and naps. In addition, the daily sleep diary also prompts caregivers to respond to questions about their child's caffeine intake, exercise, medications, and mood the previous day, as well as their routine before bed. For each rest period (nighttime or nap), the actogram will be visually inspected to locate the point where activity count drops significantly and compared with the sleep diary to set the start time of rest interval. Similarly, to mark the end of rest interval, the actogram will be inspected to locate the point where activity count rises and again compared with the sleep diary.

The primary actigraphy outcome of interest is the sleep regularity index (SRI), which is a proportion of pairs of time points 24 h apart that have matching sleep/wake status (Phillips et al., 2017; Lunsford-Avery et al., 2018). The SRI was selected as the primary actigraphy outcome because it conceptually maps on to primary targets of BSM (e.g., consistent sleep schedules) and empirically, regularity has been demonstrated as a particularly important measure of sleep regulation in early development (Melegari et al., 2020; Bundgaard et al., 2018). Secondary measures derived from actigraphy will include: sleep midpoint, total sleep time (TST; minutes from sleep start to sleep end), sleep onset latency (SOL; minutes to first sleep epoch), wake after sleep onset (WASO; minutes awake between sleep start and sleep end), and sleep efficiency (SE; TST/time in bed).

#### 2.7.5 Exploratory outcomes

The clinician-rated Impairment Rating Scale (IRS) will be completed by a blinded Ph.D. level clinical psychologist assessor and will be used to assess functional impairment in seven domains (e.g., peer relations, academics). The IRS discriminates between ADHD and non-ADHD samples (Fabiano et al., 2006), is validated in preschoolers, is sensitive to treatment effects, and is widely used in clinical trials for ADHD. Psychiatric comorbidity will be assessed by the Child Behavior Checklist for Ages 1.5–5 (CBCL), which includes both internalizing (e.g., anxiety, depression) and externalizing (e.g., ADHD, ODD) symptoms (Achenbach and Rescorla, 2000). To assess general behavior problems, the 16-item Home Situations Questionnaire (HSQ) provides ratings for the severity of behavior problems in the home setting (Barkley, 1981). Good reliability and validity have been demonstrated, and the HSQ discriminates reliably between children diagnosed with ADHD and typically developing children. Sociodemographic factors, including child, caregiver, and family characteristics, will also be collected to explore other factors that might be associated with intervention response.

Parenting experience will be assessed through three measures. The Parental Stress Scale (PSS) consists of 18 items rated on a 5-point scale to assess both positive (e.g., emotional benefits) and negative (e.g., feelings of stress) aspects of parenting (Berry and Jones, 1995). The PSS has been used with a range of child populations, including ADHD, and reliability and validity are well-established (Louie et al., 2017). The Parenting Efficacy subscale of the Parenting Sense of Competence consists of seven items rated on a 6-point scale to assess feelings of efficacy about their roles as parents (Gibaud-Wallston and Wandersman, 1978; Johnston and Mash, 1989). The measure has been used with parents of children with ADHD and has demonstrated good internal consistency. In addition, positive and negative parenting practices will be measured using three subscales from the Alabama Parenting Questionnaire (Clerkin et al., 2007).

### 2.8 Data analysis

Mean/standard deviation/median/range for continuous variables and count/percent for categorical variables will be used to summarize data. Feasibility and acceptability benchmarks for Aim 1 are described above (see Sections 2.7.1 and 2.7.2). Generalized linear models with group effect (PASS vs. BPT), time (in weeks), and group by time interactions will be utilized to test differential effects on primary outcomes: clinician-rated ADHD-RS (Aim 2) and caregiver-reported CSHQ and actigraphy-measured SRI (Aim 3), along with subject-level random intercepts and slopes. Similar models will be employed to test treatment effects on the secondary outcomes including parent-rated ADHD-RS (Aim 2) and additional actigraphy-measured constructions (e.g., SOL, midpoint, TST, SE, WASO) (Aim 3). Exploratory measures of psychosocial function, parenting stress, and comorbid symptoms will be examined using similar models. An additional generalized linear model will be applied to explore mediation for the primary outcome of clinician-rated ADHD-RS (Aim 3), with caregiver-reported CSHQ or actigraphy-measured SRI as the primary predictor and group and time as the secondary predictors, along with random intercepts and slopes.

### 2.9 Ethical considerations

We perceive minimal potential risks to study participation. The study is completely voluntary and participants are informed that they are free to refuse to answer any items on the questionnaires that they do not wish to answer. They are also informed that they are free to decline participation in any procedure and can withdraw from the study at any time. Families will receive the intervention at no cost and will be compensated for their time at assessments (baseline, post-treatment, 3 month follow up). Potential risks will be minimized by carefully monitoring any potential adverse events (AEs) and following Good Clinical Practice (GCP) guidelines. Quality Assurance includes training on Standard Operating Procedures (SOPs), tracking SOP deviations, and implementation of Good Clinical Practice (GCP) in all aspects of the project. Fidelity to procedures will be accomplished by well-defined protocols, reliability training, regular meetings to avoid drift, and internal audits conducted by the university. Data collection and storage will be managed centrally in REDCap, a web-based data management system that is secured on a standalone service maintained by the university health system (Harris et al., 2009). All connections to the system occur over encrypted channels and access is limited to approved personnel. The trial was preregistered in clinical trials.gov (NCT05862727) and has been approved by the institution's Internal Review Board (IRB).

## 3 Discussion

The PASS intervention efficiently addresses behaviors across the 24-h period for young children who are at-risk for ADHD by targeting sleep regulation as a novel mechanism for improving core ADHD symptoms. This study will be among the first to examine the impact of a combined BPT-BSM intervention on both sleep and ADHD outcomes in young children using both clinician and caregivers reports of ADHD symptoms and both caregiver-report and objective measures of sleep via actigraphy. Strengths of the study design include drawing upon a shared antecedent-behavior-consequence (ABC) framework to target both daytime and nighttime behaviors, which will allow parents the opportunity to learn, consolidate and practice skills over the course of the intervention. In addition, by identifying children at-risk for ADHD, rather than requiring full diagnosis of ADHD, this approach also permits leveraging primary care relationships to connect families to support earlier than they might otherwise receive care. Utilizing a telehealth delivery format provides a convenient and scalable mode of intervention delivery. Given that active control participants will receive a gold standard behavioral therapy for ADHD, the study design allows for blinding to the active intervention, which provides the strongest possible test of PASS.

The current study has several limitations, including a restriction to English language speakers due to therapist availability, which may limit generalizability of findings. PASS is intended for preventative use in primary care with youth who display elevated ADHD symptoms but do not have significant psychiatric or developmental comorbidities (e.g., autism spectrum disorder). As such, it is anticipated that some children will have less severe ADHD symptoms than others, and it is possible that significant ADHD improvement may not be observed due to focus on an at-risk rather than diagnosed sample. Future studies may consider examining PASS effects in children with more significant ADHD profiles and/or comorbidities (e.g., autism), and/or among children considered at-risk for ADHD based on family history. In addition, future studies may benefit from including a non-treatment control group to evaluate comparative benefit of participation in PASS vs. possible improvement in ADHD symptoms due to natural maturation processes. Children's sleep patterns may vary seasonally, but these differences could not be addressed in this study given the study timeline. SRI was selected as the primary outcome as it is ideal for capturing preschool sleep patterns specifically, including measurement across 24 h (including naps; Fischer et al., 2021). However, we acknowledge that other sleep regularity metrics may also be responsive to an intervention such as PASS, and we will calculate additional actigraphy metrics such as TST, SE, and WASO. In addition, it is important to note that metrics such as intra-individual standard deviation (StDev) and interdaily stability (IS) can also be collected from the actigraphy data collected in our protocol. Finally, future larger-scale RCT studies may evaluate the impact of PASS on functioning in preschool and school settings by including a teacher report of symptom severity, as well as the impact on circadian rhythm patterns and preferences by using a morningness–eveningness scale (e.g., Werner et al., 2009).

PASS has the potential to have a long-lasting impact on ADHD symptom trajectory as well as broader mental health concerns that may co-occur with ADHD, which may support improved sleep and behavioral health as children transition to the school years. This work will lay the foundation for continued intervention refinement that may include more stringent identification of the relative effectiveness of the component intervention processes and tailoring of the intervention (e.g., for severe presentations). We anticipate that this study will provide essential data to inform a large-scale clinical trial, with the ultimate goal of improving functional outcomes among preschoolers at risk for ADHD and modifying the trajectory of this chronic condition.
